# Association Between Levels of Pre-operative Glycosylated Hemoglobin and Post-operative Surgical Site Infections After Elective Surgery in a Low-Income Country

**DOI:** 10.7759/cureus.27397

**Published:** 2022-07-28

**Authors:** Kanza Mirza Maqsood, Ritesh Pahwani, FNU Avinash, Muhammad Raffey Shabbir, Maleeha Ali Basham, Azeem Khalid, Fizra Balkhi, Dua Khalid, Maha Jahangir

**Affiliations:** 1 General Surgery, Jinnah Postgraduate Medical Centre, Karachi, PAK; 2 Internal Medicine, Jinnah Sindh Medical University, Karachi, PAK; 3 General Surgery, Liaquat National Hospital and Medical College, Karachi, PAK; 4 Internal Medicine, Allama Iqbal Medical College, Lahore, PAK; 5 Internal Medicine, Northwell Health, Port Jefferson, USA; 6 Internal Medicine, Dow University of Health Sciences, Karachi, PAK

**Keywords:** random blood sugar (rbs), elective surgery, surgical site infection(ssi), glycated hemoglobin (hba1c), diabetes

## Abstract

Introduction: Diabetic patients undergoing surgery are more susceptible to hospital-acquired infection, particularly surgical site infection (SSI). Good glycemic control in preoperative patients significantly decreases the risk of SSI. There is a scarcity of data from low-income countries studying the relation between perioperative glycated hemoglobin (HbA1c) levels and postoperative SSI. We aim to establish statistical relation between HbA1c and SSI which will help decrease post-operative infections and morbidity.

Methods: This study was conducted in the surgical unit of Jinnah Sindh Medical University, Karachi, Pakistan, from August 2020 to April 2022. Patients who underwent elective surgical procedures (n= 1024) were included in the study and divided into two groups based on their HbA1c levels. Patients with HbA1c levels higher than 6.5% were classified as group A and those with HbA1c less than 6.5% belonged to group B. For statistical analysis, IBM SPSS Statistics for Windows, Version 24.0 (Released 2016; IBM Corp., Armonk, New York, United States) was used.

Results: Group A comprised 579 (56.5%) patients. The presence of SSI in participants with HbA1c >6.5% was statistically significant (p-value: 0.011). Genderwise comparison with the presence of SSI was found to be insignificant (p-value: 0.28). Smoking was positively correlated with the absence of SSI. No significance in terms of presence or absence of SSI was found in the comparison of the type of wounds (p-value: 0.25).

Conclusion: There is a positive relationship between raised HbA1c levels and the development of SSI. Our study emphasizes the importance of the use of HbA1c levels as a more accurate predictor of glycemic control in pre-operative patients rather than blood glucose levels. It is imperative that surgeons must check HbA1c levels before selecting patients for elective surgeries, especially in low-income countries where the healthcare burden is already huge.

## Introduction

Surgical site infection (SSI) is an infection that occurs at or near surgical incisions. The Centers for Disease Control and Prevention (CDC) classifies SSI into incisional (superficial or deep) or organ space [1]. There are several independent risk factors of SSI that increase the mortality and morbidity among surgical patients including the type of procedure, type of wound, preoperative antiseptic techniques, diabetes, hypertension, cigarette smoking, obesity, blood loss, hypothermia, and native flora of patient’s own skin or visceral [2].

Patients undergoing surgery with hyperglycemia due to type 2 diabetes mellitus (DM) are more susceptible to hospital-acquired infection [3]. In recent years, clinical trials and studies evaluated that blood glucose level control in preoperative patients significantly decreases mortality and morbidity [3]. In 2021, an explorative analysis in patients undergoing surgery was conducted where the role of glycated hemoglobin (HbA1c) as a positive perioperative predictor for SSI was discussed. About 38.5% of infections were found among patients with undiagnosed and pre-diabetic states [4]. Ahmed M et al. also conducted a cross-sectional study with a sample size of 163 patients where patients with more than 8.5% HbA1c were vulnerable to superficial SSI (33.1%) and deep SSI (12.3%). Therefore, he concluded that HbA1c is a proper modifiable independent risk factor for SSI [5].

It is of significant value to determine the scenarios and subgroups of diabetic individuals where preoperative HbA1c plays role in predicting the risks of increased post-operative complications [6]. There is a scarcity of data from low-income countries studying the relation between perioperative HbA1c levels and post-operative SSI and its overall impact. We aim to fill the gap of this lack of statistical relation between HbA1c and SSI which will help decrease post-operative morbidity and mortality, length of hospital stay, and readmissions, leading to a better quality of life.

## Materials and methods

This cohort study was conducted in the surgical unit of Jinnah Sindh Medical University (JSMU), Karachi, Pakistan, from August 2020 to April 2022 after taking ethical review board approval (JSMU/IRB/2020-81). Patients who underwent elective surgical procedures, such as appendectomy, cholecystectomy, and laparotomy during this time frame were enrolled in the study via consecutive convenient non-probability sampling after taking their informed consent.

After enrollment, as part of the pre-operative assessment, patients' blood was taken via phlebotomy via a cubital vein and sent to the laboratory for random blood sugar (RBS) and HbA1c, along with a complete blood report. Patients with elevated RBS were excluded from the study. Only patients operated by the same surgeon in the same operating room were included in the study to reduce the impact on an individual's skill and the environment. After exclusion, 1024 participants were included in the study. Patients were divided into two groups based on their HbA1c levels. Patients with HbA1c levels higher than 6.5% were classified as group A and patients with HbA1c levels lower than 6.5% were classified as group B.

All surgical procedures were carried out in accordance with protocol. Following a review of the operational reports, each surgery received one of four wound classifications, i.e. clean, clean/contaminated, contaminated, or dirty. Patients were instructed to return for a check-up following the treatment after one week, then again after 30 days, or if they experienced any redness, leaking, pain, or tenderness at the site of the surgical incision. Based on documented purulence, discomfort, redness, tenderness, swelling, and suspicion of SSI upon physical examination, patients were categorized as having SSI or not during follow-up. The final analysis excluded participants who could not be reached for follow-up.

For statistical analysis, IBM SPSS Statistics for Windows, Version 24.0 (Released 2016; IBM Corp., Armonk, New York, United States) was used. While categorical data was shown as frequency and percentage, continuous variables were assessed using descriptive statistics and displayed as means ± standard deviations (SDs). To compare the two groups, the chi-square test was used. When the p-value is less than 0.05, the null hypothesis is rejected since there is a significant difference between the groups.

## Results

Of the total 1024 participants included in the final analysis, 579 (56.5%) belonged to group A while the remaining belonged to group B. Mean age of patients from group A was 43 ± 8 years while 45 ± 8 years of group B (p-value: 0.0001). There were no statistical differences between the two groups in terms of gender (p-value: 0.88), and other comorbidities, including smoking (p-value: 0.306) and hypertension (p-value: 0.77). A majority of participants had a clean type of wound in both groups A and B (95.5% and 95.9%), however, no significant relation was found while comparing the types of wound (p-value: 0.85). The presence of SSI in participants with HbA1c >6.5% was statistically significant (p-value: 0.011) (Table 1).

**Table 1:** Comparison of characteristics in both groups SD: standard deviation, SSI: surgical site infection

Demographics	Group A (n=579)	Group B (B=445)	p-value
Age (years) (Mean ± SD)	43 ± 8	45 ± 8	0.0001
Gender			
Male	302 (52.1%)	230 (51.6%)	0.88
Female	277 (47.9%)	215 (48.4%)	
Comorbidities			
Smoker (%)	105 (18.1%)	92 (20.6%)	0.306
Hypertension (%)	212 (36.6%)	159 (35.7%)	0.77
Type of wounds			
Clean	553 (95.5%)	426 (95.9%)	0.85
Clean/Contaminated	23 (3.9%)	18 (4.0%)	
Contaminated	1 (0.1%)	2 (0.4%)	
Dirty	2 (0.3%)	1 (0.2)	
Overall SSI	69 (11.9%)	32 (7.19%)	0.011

Genderwise comparison with the presence of SSI was found to be insignificant (p-value: 0.28). Smoking was positively correlated with the absence of SSI (p-value: <0.00001). Overall more participants of group A had clean type of wound (n=94), followed by clean contaminated (n=5). Similarly, in group B, 883 participants had clean while 36 participants had a clean contaminated type of wound. No significance was found on the comparison of the type of wounds (p-value: 0.25) (Table 2).

**Table 2:** Comparison of characteristics with the presence and absence of SSI SSI: surgical site infection

Demographics	SSI present (n=101)	SSI absent (n=923)	p-value
Gender			
Male (n=532)	55 (10.3%)	477 (89.6%)	0.28
Female (n=492)	46 (9.3%)	446 (90.7%)	
Comorbidities			
Smoker (n=197)	62 (31.4%)	137 (68.6%)	<0.00001
Hypertension (n=371)	39 (10.4%)	332 (89.6%)	0.59
Type of wounds			
Clean (n=977)	94 (9.6%)	883 (90.4%)	0.25
Clean/Contaminated (n=41)	5 (12.1%)	36 (87.9%)	
Contaminated (n=3)	1 (33.3%)	2 (66.6%)	
Dirty (n=3)	1 (33.3%)	2 (66.6%)	

## Discussion

Prevention of surgical site infections is important since it plays a significant role in hospital readmissions, prolonged stays, and greater financial burden [7]. After doing thorough literature research, we came to the conclusion that many studies support the association between elevated HbA1c levels with increased risk for SSI [8-10]. This aligns with the findings of our study where patients with HbA1c levels greater than 6.5% were more likely to develop SSI as compared to those who had lower levels (p-value: 0.01). A study done by Kopp Lugli et al. found an overall 4.1 times increased risk of developing SSI [4]. Raised HbA1c preoperatively is linked with an increased risk of infection, length of hospital stay and readmission in 30 days [9]. 

However, it is worth mentioning that we also came across a few studies that either showed mixed results or did not find any conclusive relationship between HbA1c and SSI [11]. In a 2019 study, the probability of developing surgical complications or readmission with respect to gender, age, or presence of hypertension was found to be nonsignificant [9]. Furthermore, no association between the type of wound and HbA1c levels was found in our study. In our study, we used HbA1c rather than fasting blood sugar (FBS) or RBS levels because HbA1c is a more accurate marker of glycemic control in comparison to RBS or FBS; levels of which can rise due to stress hyperglycemia in pre-operative patients. Furthermore, even though candidates in our study had normal FBS/RBS levels before surgery, the group with HbA1c levels greater than 6.5% had a higher chance of developing SSI.

HbA1c is a marker of variation in blood glucose levels. Fluctuation in glucose levels causes an increased production of free radicals which generates increased oxidative stress. In a study, it was established that acute and chronic fluctuations in blood glucose levels corresponded with elevated oxidative stress markers namely: urine 8-isoprostaglandin F2α, serum thiobarbituric acid-reactive substance, and serum 8-hydroxydeoxyguanosine [9,12]. In addition, variation in glucose levels also induced a rise in serum level of the chronic inflammatory marker (c-reactive protein), absence of antioxidants, and a greater incidence of microvascular complications [12-15]. Other than increasing oxidative stress and inflammatory markers, hyperglycemia also harms the immune system by disrupting chemotaxis, phagocytosis, and overproduction of free fatty acids [16]. All these factors altogether may lead to an increased risk of developing SSI.

Our study had some limitations, it was a single-center study, and patients had different procedures, different types, and sites of wounds. We used a single cut-off value; a different cut-off might have yielded a different result. The use of HbA1c rather than FBS or RBS brought accuracy to our results as it eliminated the probability of patients having stress hyperglycemia. We also made sure our results were not affected by surgeon skill and the environment by choosing patients operated on by the same surgeon in the same operating room.

## Conclusions

Correlating the results of our study with preexisting literature, our study is of the view that there is a positive relationship between raised HbA1c levels and the development of SSI. Furthermore, our study emphasizes the importance of the use of HbA1c levels as a more accurate predictor of glycemic control in pre-operative patients rather than blood glucose levels. Since SSI plays a significant role in patient morbidity, mortality, and financial burden, it is imperative that surgeons must check HbA1c levels before selecting patients for elective surgeries, especially in low-income countries where the healthcare burden is already huge.
